# Analysis of the impact of determinants of kosherness on the content of macro‐ and microelements in beef

**DOI:** 10.1002/fsn3.1192

**Published:** 2019-09-10

**Authors:** Mariusz Rudy, Jagoda Żurek, Renata Stanisławczyk, Marian Gil, Paulina Duma‐Kocan, Grzegorz Zaguła, Stanisław Rudy

**Affiliations:** ^1^ Chair of Agricultural Processing and Commodity Science Department of Biology and Agriculture University of Rzeszow Rzeszów Poland; ^2^ Poultry Processing Plant Rzeszowskie Zakłady Drobiarskie RES‐DROB Rzeszów Poland; ^3^ Chair of Bioenergetics and Food Analysis Department of Biology and Agriculture University of Rzeszow Rzeszów Poland; ^4^ Department of Thermal Technology and Food Process Engineering Faculty of Production Engineering University of Life Sciences Lublin Poland

**Keywords:** beef, bulls, heifers, ICP‐OES, minerals, ritual slaughter

## Abstract

There are no studies in the literature that comprehensively present the impact of factors related only to kosher slaughter on the content of minerals in beef. Therefore, learning the impact of such kosher determinants of beef as follows: no stunning during slaughter, cattle category, muscle, or the so‐called kosher treatment for mineral content in beef has an original character. In this connection, the aim of the research was a comprehensive analysis of the impact of kosher determinants (slaughter type, muscle, cattle category, and technological treatment) on the content of selected macro‐ and microelements in beef. On the content of minerals in muscles obtained from beef carcasses, the statistically significant (*p* < .05) impact was found in the case of slaughter type for such elements as: K, P, and Na. Higher content of phosphorus, potassium, and sodium was found in muscles of heifers and bulls from standard slaughter, compared with the amount of these elements determined in muscles of cattle from kosher slaughter. In turn, statistically significant (*p* < .05) impact of cattle sex was confirmed only in the case of iron and molybdenum content in beef. Higher amounts of these elements were determined in muscle from heifer carcasses (excluding the molybdenum content in MLT muscle of heifer carcasses from kosher slaughter). The process of koshering (soaking in brine) causes approximately 10‐fold increase in the amount of sodium in beef, regardless of the muscle or gender of cattle. The statistically significant impact of muscle was confirmed only in the case of zinc content. In the authors’ own research, there was found no statistically significant impact of the interaction effects between the analyzed factors (S × G, S × M, G × M, and S × G × M) on the content of particular mineral components.

## 1 INTRODUCTION

Depending on the breed, muscle type, diet, and processing, the content of micro‐ and macroelements in meat is varied (Cabrera, Ramos, Saadoun, & Brito, 2010; Chen, Pearson, Gray, Fooladi, & Ku, 1984; Hintze, Lardy, Marchello, & Finley, 2001; Purchas & Busboom, 2005; Purchas, Simcock, Knight, & Wilkinson, 2003; Revilla & Vivar‐Quintana, 2006). Bioavailability of selected ingredients in meat is much higher than in foods of plant origin (Biesalski & Nohr, 2009; Troy & Kerry, 2010). Bioavailability of iron from meat is almost twice as high as from plant products. A similar situation applies to absorption of zinc when using a diet rich in animal proteins (Mulvihill & Morrissey, 1998). Bioavailability of elements of meat oscillates between 55% and 95% for manganese, 60 and 70% for iron, 40 and 68% for zinc, and 30 and 45% for copper (Ramos, Cabrera, & Saadoun, 2012).

In the world, there are many methods of slaughtering animals which originate from different religions and cultures. The principles of kosher nutrition of Jews are based on the commandments contained in the Torah, interpreted and perfected by Jewish religious leaders (Regenstein & Regenstein, 1991). The main requirements of kosher slaughter commonly used by Jews include: vitality and awareness of animals slaughtered. Stunning is unacceptable. In addition, kosherness requires removal of flowing blood considered as impurity which should not be consumed.

Ritual slaughter causes the most effective bleeding of carcases, which, in terms of quality and hygiene, best suits human needs. Meat from ritual slaughter is more durable and does not spoil quickly—it contains less blood and more nutrients (Dembo, 2016).

In the professional literature, there is information on the specification of various types of ritual slaughter of cattle or other animal species (Farouk, 2013; Farouk et al., 2014; Velarde et al., 2014). For example, kosher slaughter, as compared with non‐kosher one, reduces meat pH (Holzer, Berry, Campbell, Spanier, & Solomon, 2004). In addition, koshering removes myoglobin and other sarcoplasmic proteins as a result of soaking the raw material in cold water (30 min) and salting the surface with kosher coarse salt (about 1 hr) (Regenstein & Regenstein, 1988). The removal of myoglobin causes changes in color, taste, and overall quality of the product, but from the point of view of health, the most important effect is its impact on oxidation processes (Baron, Skibsted, & Andersen, 1997; Lapidot, Granit, & Kanner, 2005). However, reducing the amount of heme proteins affects the color of the final product. Kosher meat has been shown to have low color intensity (Holzer et al., 2004). In addition, a high salt content (Mast & Macneil, 1983) is an important factor in the nutritional quality of kosher meat, compared with the meat obtained from standard slaughter.

However, there are no studies in the literature that comprehensively present the impact of factors related only to kosher slaughter on the content of minerals in beef. Therefore, learning the impact of such kosher determinants of beef as follows: no stunning during slaughter, cattle category, muscle, or the so‐called kosher treatment for mineral content in beef has an original character. In this connection, the aim of the research was a comprehensive analysis of the impact of kosher determinants (slaughter type, muscle, cattle category, and technological treatment) on the content of selected macro‐ and microelements in beef.

## 2 MATERIALS AND METHODS

### 2.1 Materials

The research material was muscle: the longest thoracic muscle *musculus longissimus thoracis*—MLT and supraspinatus (*musculus supraspinatus*—MS) obtained from 40 carcasses of heifers and 40 carcasses of bulls (up to 24 months old), coming from crossing of cows of the Polish Holstein‐Friesian breed with bulls of the Limousine breed. The animals were kept in a semi‐intensive system. The basic animal feed during the summer period was green matter of grasses and maize silage, while during the winter period it was maize silage. A supplement of the dose was meadow hay and ground grain. The cattle, after being brought to one of meat plants in southeastern Poland, was kept in a livestock warehouse in single pens for 20 hr, and then, after weighing, the animals were slaughtered in accordance with the methodology used in meat industry.

The animals came from two types of slaughter:

- standard with mechanical stunning with a pneumatic captive bolt pistol (40 heads);
- ritual kosher—without stunning, combined with the so‐called koshering, which is carried out after about 24 hr of cooling down of carcasses after slaughter, and consists in preliminary rinsing quarters in water with specified parameters, then salting and rewashing 3 times (40 heads).

The carcasses were not subjected to electrostimulation, and after the assessment according to the EUROP system, they were qualified in terms of forming as the following classes: O—(50%) and R—(50%), while in terms of fatness as class 3 (100%).

### 2.2 Sampling and instrumental analysis

Forty‐eight hours after slaughter, 50 g of samples was collected from each muscle in cold storage (0–2°C), which were purified from fat and connective tissue, and then broken up and homogenized. The homogenized fresh meat samples were subjected to microwave mineralization under high‐pressure in a super pure 65% HNO_3_. For this purpose, a 0.5 g sample (in three replications) was placed in Teflon vessels, which were then filled up with 8 ml of nitric acid and sealed hermetically.

For each group of nine samples, the rotor of a digestion system was also filled with a blank sample. The samples were digested at an algorithm of temperature increasing as specified for biological samples, never exceeding 200ºC. This procedure was carried out in an Ethos One microwave digestion system from Milestone. The vessels were opened after the mineralization process had been completed and the samples with acid had been brought to room temperature. Afterward, they were replenished with water to a volume of 50 ml. The detection threshold obtained for each element was not lower than 0.01 mg/kg (with an assumed detection capacity of the measuring apparatus at a level exceeding 1 ppb). The measurements were performed on an ICP‐OES spectrometer, Thermo iCAP Dual 6,500 with horizontal plasma, and with the capacity of detection being determined both along and across the plasma flame (Radial and Axial). Before measuring each batch of 10 samples, the equipment was calibrated with the use of certified Merck models, with concentrations of 10,000 ppm for Ca, Fe, K, Mg, and P and 1,000 ppm for Al, Cr, Cu, Mn, Mo, Na, Ni, S, and Zn. The measurement result for each element was adjusted to account for the measurement of elements in the blank sample.

In each case, a 3‐point calibration curve was used for each element, with optical correction applying the method of internal models, in the form of yttrium and ytterbium ions, at concentrations of 2 mg/L and 5 mg/L, respectively. The analytical methods were validated using two independent tests.

The Certified Reference Material was used when obtaining recoveries for individual elements, as in Table 1. However, for identification of appropriate slotted lines and avoidance of possible interferences, the method of standard addition with known concentration was applied (recovery results in Table 1). The detection limit for each element was determined at a level not lower than 1 µg/L.

**Table 1:** The lengths of measurement lines and the recovery obtained for the specific elements examined

No.	Element	Slotted line (nm)	Recovery according to CRM (%)	Recovery according to method of standard addition (%)
1	Al	167.1	98	99
2	Ca	393.4	103	98
3	Cr	267.7	99	97
4	Cu	324.8	99	97
5	Fe	259.9	101	97
6	K	766.5	99	98
7	Mg	279.6	98	97
8	Mn	257.6	101	99
9	Mo	202	98	100
10	Na	589.6	99	100
11	Ni	231.6	100	101
12	P	177.5	101	98
13	S	180.7	103	99
14	Zn	202.5	100	100

### 2.3 Statistical analyses

All analyzes were performed in triplicate. The obtained results were grouped and subjected to statistical calculations. Data were analyzed using a three‐way analysis of variance to determine differences in contents in beef of elements, which were influenced by the type of slaughter, gender, and muscle. The main effects of the type of slaughter, gender, muscle, and their reactions were determined using the GLM procedure (ANOVA; STATISTICA v. 13.1; StatSoft, Krakow, Poland) for a fixed‐effect model with 2 slaughter types, 2 sex groups, and 2 muscle groups. When the effects were significant (*p* < .05), the averages were compared with the *post hoc* HSD Tukey's test (ANOVA, STATISTICA v. 13.1; StatSoft). Table 2 presents the average values and standard errors (*SE*) of the contents of the elements determined in beef meat. To determine the relationship between the indicated elements, the correlation coefficients of Pearson's (*r*) line were calculated. Tables 3, 4 contain correlation coefficients. Values of correlation coefficients were interpreted according to the following scale: *r* ≤ 0.2 unclear correlation, 0.2–0.4 clear but weak correlation, 0.4–0.7 moderate correlation, 0.7–0.9 strong correlation, and *r* > 0.9 very strong correlation.

**Table 2:** The content of minerals (mg/100 g) in muscles from cattle carcasses (*n* = 80)

Specification	Gender	Standard slaughter		Kosher slaughter		ANOVA
		MLT (*n* = 40) Mean ± *SE*	MS (*n* = 40) Mean ± *SE*	MLT (*n* = 40) Mean ± *SE*	MS (*n* = 40) Mean ± *SE*	
K	B	350.39^a^ ± 3.98	335.83 ± 5.47	299.34 ± 13.19	298.06 ± 8.67	S*
	H	346.69^a^ ± 4.99	317.52 ± 5.59	284.62^b^ ± 14.20	293.29^b^ ± 10.63	
P	B	197.86^a^ ± 1.59	195.08^a^ ± 2.50)	183.39^b^ ± 3.51	184.68^b^ ± 3.17	S*
	H	200.47^a^ ± 2.54	193.05^a^ ± 1.63	182.19^b^ ± 5.93	188.53^b^ ± 4.00	
Na	B	46.68^a^ ± 1.98	54.82^a^ ± 2.30	469.28^b^ ± 9.09	418.31^b^ ± 11.79	S*
	H	40.27^a^ ± 1.70	49.82^a^ ± 2.50	606.78^b^ ± 11.98	447.82^b^ ± 7.83	
Mg	B	27.66 ± 0.41	27.33 ± 0.44	31.41 ± 0.48	23.73 ± 0.58	–
	H	27.02 ± 0.56	25.30 ± 0.36	23.17 ± 0.96	24.46 ± 0.70	
Fe	B	1.31^c^ ± 0.01	1.49^c^ ± 0.15	1.21^c^ ± 0.17	1.37^c^ ± 0.17	G*
	H	1.59^d^ ± 0.15	1.80^d^ ± 0.18	1.52^d^ ± 0.17	2.66^d^ ± 0.19	
Ca	B	5.53 ± 0.62	4.71 ± 0.46	11.70 ± 0.33	8.15 ± 0.87	–
	H	5.59 ± 0.55	4.72 ± 0.29	6.41 ± 0.60	6.18 ± 0.56	
S	B	191.58 ± 1.11	194.08 ± 2.38	173.41 ± 10.40	186.19 ± 1.98	–
	H	193.91 ± 2.15	192.25 ± 2.03	186.91 ± 3.84	196.44 ± 3.17	
Zn	B	2.51^x^ ± 0.08	3.59^y^ ± 0.22	2.80^x^ ± 0.23	3.60^y^ ± 0.25	M*
	H	2.73^x^ ± 0.15	3.56^y^ ± 0.14	2.65^x^ ± 0.10	3.90^y^ ± 0.18	
Cu	B	0.03 ± 0.0002	0.05 ± 0.0022	0.04 ± 0.0013	0.06 ± 0.0022	–
	H	0.03 ± 0.0004	0.03 ± 0.0002	0.02 ± 0.0004	0.04 ± 0.0002	
Mn	B	0.03 ± 0.0002	0.04 ± 0.0022	0.09 ± 0.045	0.03 ± 0.0022	–
	H	0.04 ± 0.0004	0.03 ± 0.0004	0.03 ± 0.0002	0.04 ± 0.0002	
Mo	B	0.000467^c^ ± 0.00003	0.000200^c^ ± 0.00003	0.000933 ± 0.00003	0.000867^c^ ± 0.00002	G*
	H	0.002133^d^ ± 0.00005	0.001200^d^ ± 0.00003	0.000900 ± 0.00003	0.001667^d^ ± 0.00005	
Al	B	0.02 ± 0.0009	0.03 ± 0.002	0.08 ± 0.0036	0.02 ± 0.001	–
	H	0.004700 ± 0.0002	0.02 ± 0.001	0.04 ± 0.0022	0.00 ± 0.00	
Ni	B	0.06 ± 0.002	0.03 ± 0.0022	0.03 ± 0.002	0.01 ± 0.0004	–
	H	0.03 ± 0.002	0.02 ± 0.0007	0.01 ± 0.001	0.03 ± 0.0009	
Cr	B	0.06 ± 0.0016	0.02 ± 0.0007	0.08 ± 0.0029	0.02 ± 0.0007	–
	H	0.03 ± 0.0007	0.01 ± 0.0004	0.03 ± 0.0004	0.04 ± 0.0007	

Note

Various superscript letters (a, b) at averages mean statistically significant differences (*p* < .05) due to the effect of type of slaughter. Various superscript letters (c, d) at averages mean statistically significant differences (*p* < .05) due to the effect of gender. Various superscript letters (x, y) at averages mean statistically significant differences (*p* < .05) due to the effect of muscle. ANOVA—three‐way ANOVA analysis among the type of slaughter, S; muscle, M; gender, G.

Abbreviations: B, bulls; H, heifers.

* *p* < .05.

**Table 3:** Correlation coefficients between the analyzed minerals determined in muscles of cattle carcasses from standard slaughter (*n* = 40)

Specification	K	P	Na	Mg	Fe	Ca	S	Zn	Cu	Mn	Mo	Al	Ni	Cr
K	–	0.834*	−0.417	0.781*	0.073	0.227	0.374	−0.470	−0.057	0.132	0.096	0.103	0.484	0.368
P	0.834*	–	−0.215	0.840*	0.206	0.259	0.588*	−0.365	0.008	0.251	0.178	0.084	0.458	0.284
Na	−0.417	−0.215	–	−0.019	0.142	0.081	0.218	0.558*	0.237	−0.004	−0.216	−0.029	−0.050	−0.078
Mg	0.781*	0.840*	−0.019	–	0.239	0.315	0.581*	−0.265	0.244	0.069	−0.168	0.310	0.472	0.308
Fe	0.073	0.206	0.142	0.239	–	0.146	0.505*	0.379	0.377	0.208	0.132	0.206	0.226	−0.007
Ca	0.227	0.259	0.081	0.315	0.146	–	0.208	0.020	−0.028	−0.163	−0.060	−0.104	0.289	0.317
S	0.374	0.588*	0.218	0.581*	0.505*	0.208	–	0.242	0.279	0.164	0.106	0.079	0.133	0.223
Zn	−0.470	−0.365	0.558*	−0.265	0.379	0.020	0.242	–	0.362	−0.079	−0.006	0.069	−0.269	−0.175
Cu	−0.057	0.008	0.237	0.244	0.377	−0.028	0.279	0.362	–	0.037	−0.162	0.520*	0.125	−0.137
Mn	0.132	0.251	−0.004	0.069	0.208	−0.163	0.164	−0.079	0.037	–	0.466	−0.208	0.404	0.185
Mo	0.096	0.178	−0.216	−0.168	0.132	−0.060	0.106	−0.006	−0.162	0.466	–	−0.202	0.174	0.179
Al	0.103	0.084	−0.029	0.310	0.206	−0.104	0.079	0.069	0.520*	−0.208	−0.202	–	0.136	−0.005
Ni	0.484	0.458	−0.050	0.472	0.226	0.289	0.133	−0.269	0.125	0.404	0.174	0.136	–	0.378
Cr	0.368	0.284	−0.078	0.308	−0.007	0.317	0.223	−0.175	−0.137	0.185	0.179	−0.005	0.378	–

* Correlation coefficient statistically significant at the level of *p* < .001.

**Table 4:** Correlation coefficients between the analyzed minerals determined in muscles of cattle carcasses from kosher slaughter (*n* = 40)

Specification	K	P	Na	Mg	Fe	Ca	S	Zn	Cu	Mn	Mo	Al	Ni	Cr
K	–	0.899*	−0.709*	−0.068	0.005	−0.490	0.566*	−0.075	−0.021	−0.350	0.525*	−0.134	−0.044	−0.230
P	0.899*	–	−0.632*	0.068	0.110	−0.348	0.549*	−0.091	0.073	−0.210	0.473	−0.041	0.072	−0.077
Na	−0.709*	−0.632*	–	−0.344	−0.048	0.062	0.017	0.341	−0.375	−0.144	−0.244	−0.147	−0.200	−0.051
Mg	−0.068	0.068	−0.344	–	−0.029	0.869*	−0.704*	−0.449	0.581*	0.945*	0.024	0.736*	0.717*	0.615*
Fe	0.005	0.110	−0.048	−0.029	–	−0.069	0.126	0.097	0.313	−0.004	0.144	−0.017	0.109	0.038
Ca	−0.490	−0.348	0.062	0.869*	−0.069	–	−0.831*	−0.305	0.466	0.951*	−0.188	0.718*	0.657*	0.594*
S	0.566*	0.549*	0.017	−0.704*	0.126	−0.831*	–	0.463	−0.452	−0.844*	0.424	−0.552*	−0.396	−0.458
Zn	−0.075	−0.091	0.341	−0.449	0.097	−0.305	0.463	–	−0.222	−0.412	0.227	−0.379	−0.215	−0.156
Cu	−0.021	0.073	−0.375	0.581*	0.313	0.466	−0.452	−0.222	–	0.534*	−0.079	0.406	0.404	0.311
Mn	−0.350	−0.210	−0.144	0.945*	−0.004	0.951*	−0.844*	−0.412	0.534*	–	−0.109	0.709*	0.675*	0.623*
Mo	0.525*	0.473	−0.244	0.024	0.144	−0.188	0.424	0.227	−0.079	−0.109	–	−0.012	0.306	−0.037
Al	−0.134	−0.041	−0.147	0.736*	−0.017	0.718*	−0.552*	−0.379	0.406	0.709*	−0.012	–	0.715*	0.486
Ni	−0.044	0.072	−0.200	0.717*	0.109	0.657*	−0.396	−0.215	0.404	0.675*	0.306	0.715*	–	0.532*
Cr	−0.230	−0.077	−0.051	0.615*	0.038	0.594*	−0.458	−0.156	0.311	0.623*	−0.037	0.486	0.532*	–

* Correlation coefficient statistically significant at the level of *p* < .001.

## 3 RESULTS

On the content of minerals (mg/100 g) in muscles obtained from beef carcasses (Table 2), the statistically significant (*p* < .05) impact was found in the case of slaughter type for such elements as follows: K, P, and Na. Higher potassium content (*p* < .05) was found in MLT muscles of heifers and bulls from standard slaughter, compared with the amount of this element determined in muscles of heifer carcasses from kosher slaughter. Higher content of phosphorus (*p* < .05) was found in muscles of heifers and bulls from standard slaughter, compared with the amount of this element determined in muscles of cattle from kosher slaughter. On the other hand, about 10 times higher sodium content (*p* < .05) was determined in muscles of cattle from kosher slaughter. In turn, statistically significant (*p* < .05) impact of cattle sex was confirmed only in the case of iron and molybdenum content in beef. Higher amounts of these elements (*p* < .05) were determined in muscle from heifer carcasses (excluding the molybdenum content in MLT muscle of heifer carcasses from kosher slaughter). The statistically significant impact of muscle was confirmed only in the case of zinc content. Higher amounts of this element (*p* < .05) were determined in the MS muscle of cattle, regardless of the type of slaughter. In the authors’ own research (Table 2), there was found no statistically significant (*p* < .05) impact of the interaction effects between the analyzed factors (S × G, S × M, G × M, and S × G × M) on the content of particular mineral components.

Considering the correlation coefficients between the analyzed mineral components determined in muscles of cattle carcasses from standard slaughter (Table 3) and kosher slaughter (Table 4), it should be noted that kosher slaughter compared with a standard one causes an increase in the number of pairs of statistically significant relationships (*p* < .001) from 8 to 26. Among statistically significant (*p* < .001) dependencies that were found between the analyzed elements determined in muscles of cattle from kosher slaughter can be mentioned:

Positive correlations between the content of:

- K and the amount S and Mo;
- Mg and the amount of Ca, Cu, Mn, Al, Ni, and Cr;
- Ca and the amount of Mn, Al, Ni, and Cr;
- Cu and the amount of Mn;
- Mn and the amount of Al, Ni, and Cr;
- Al and the amount of Ni.

Negative coefficients between the content of:

- K and the amount of Na;
- P and the amount of Na;
- Ca and the amount of S;
- S and the amount of Mn and Al

It should also be noted that the correlation coefficients between the same elements determined in muscles of cattle carcasses from standard slaughter were statistically insignificant. However, among statistically significant relationships (*p* < .001) which, in turn, were found between the elements determined in muscles of cattle carcasses from standard slaughter (coefficients of correlation between the same elements determined in muscles of cattle from kosher slaughter were statistically insignificant) can be mentioned:

Positive correlations between the content of:

- K and the amount of Mg;
- P and the amount of Mg;
- Na and the amount of Zn;
- Fe and the amount of S;
- Cu and the amount of Al.

## 4 DISCUSSION

Consumption of red meat may be a good way to cover the body's demand for macro‐ and microelements (Forestell, Spaeth, & Kane, 2012; McNeill & Van Elswyk, 2012; Williams, 2007; Williamson, Foster, Stanner, & Buttriss, 2005). The concentration of nutrients in individual elements of meat can be influenced, among others, by differences in histology, functions performed in the body, and intensity of work during life of the animal (Cabrera et al., 2010; Driskell et al., 2011; García‐Vaquero, Miranda, Benedito, Blanco‐Penedo, & López‐Alonso, 2011; Lawrie & Ledward, 2006; Ramos et al., 2012). According to Kołczak (2008), the content of minerals in culinary beef is about 1%.

Beef is considered as a rich source of iron and zinc, with high bioavailability. Literature data, however, indicate a large variation in the content of these nutrients in beef (Cabrera et al., 2010; García‐Vaquero et al., 2011; Goran, Tudoreanu, Rotaru, & Crivineanu., 2016; Kerry & Ledward, 2009; Ramos et al., 2012; Williams, 2007; Williamson et al., 2005).

Menezes, Oliveira, Franca, Souza, and Nogueira (2018) showed that the values of Ca (105 mg/kg), Cu (1.9 mg/kg), Fe (49 mg/kg), Mg (763 mg/kg), and Zn (172 mg/kg) in raw beef are higher than in the analyzed samples, except for the level of Ca in MLT muscle of bulls from kosher slaughter, the value of which was 11.70 mg/100 g. The contents of Ca determined by Gerber et al. (Gerber, Scheeder, & Wnek, 2009) in beef elements (5.4–7.0 mg/100 g) are at a similar level as in the author's own research.

Thermal processing of meat can lead to significant modifications of macro‐ and micromineral levels, which depend on the process and type of cooking (Gerber et al., 2009; Lombardi‐Boccia, Lanzi, & Aguzzi, 2005). Menezes et al. (2018) found that among various heat treatments, cooking resulted in the highest percentage of Ca bioavailability, while the lowest results were observed for samples cooked at 180°C for 45 min and for raw samples. The author's own research confirmed that meat is not the main source of calcium.

Gerber et al. (2009) observed increase in Fe content after heat treatment of beef samples. In addition, the cited authors assessed the effect of heat treatment on high‐fat meat elements and showed that the concentrations of Ca, Na, K, Mg, and P decreased after cooking.

In the body of animal, iron accumulates in the liver (Murray et al., 2012). Duhaiman (1988) and Franco (1992) showed that Fe content in bovine liver is higher than in muscles. Similar relations in iron content between muscles and liver were described by Valenzuela, Romaña, Olivares, Morales, and Pizarro (2009). In addition, López Alonso et al. (2004) found that iron content in beef liver may vary from 3.7 to 22.3 mg/100 g.

From among beef, pork, and poultry, the first of them can be considered the main source of bioactive Fe (Menezes et al., 2018). Pretorius, Schönfeldt, and Hall (2016) and Czerwonka and Szterk (2015) found that the level of iron (2.04–2.45 and 1.73–2.28 mg/100 g respectively) in analyzed beef elements is higher than in the author's own research, with the exception of MS muscle of heifers from kosher slaughter, in which the content of this element was determined at 2.66 mg/100 g. Gerber et al. (2009) showed that the amount of iron (1.42–1.61 mg/100 g) in beef elements is at a level similar to that in analyzed muscles in the author's own research. Lombardi‐Boccia et al. (2005) observed that the concentration of iron (1.8–2.37 mg/100 g) in particular beef elements is higher than the content of this element determined in meat of cattle in the author's own research, with the exception of MS muscles of heifers from standard slaughter (1.80 mg/100 g) and kosher slaughter (2.66 mg/100 g).

Czerwonka and Szterk (2015) and Gerber et al. (2009) showed that sodium content (48–74 mg/100 g and 45–65 mg/100 g, respectively) in beef elements is at a similar level as in the analyzed muscles of bulls and heifers from standard slaughter. Czerwonka and Szterk (2015) showed the highest concentration of this mineral in *m. infraspinatus* muscle. MLT and MS muscles of cattle from kosher slaughter showed a much higher content of this mineral, which resulted from the process of koshering of meat.

The results concerning potassium content in selected beef muscles observed by Czerwonka and Szterk (2015) were higher (381.0–430.1 mg/100 g) than the amount of this element determined in the analyzed muscles in the author's own research. The amount of potassium found in MLT and MS muscles of bulls and heifers from standard slaughter in the author's own research was at similar levels (309–337 mg/100 g), as observed in beef elements by Gerber et al. (2009). In the author's own research, in MLT and MS muscles in both genders of cattle from kosher slaughter, there was found a lower content of potassium than in meat of these animals from standard slaughter.

Czerwonka and Szterk (2015) found that the amount of magnesium in beef is at a similar level to that in the analyzed muscles in the author's own research, where the highest content of this mineral (31.41 mg/100 g) was found in MLT muscle of bulls from kosher slaughter.

In the author's own research, zinc content in the analyzed muscles was slightly lower than in beef elements determined by Gerber et al. (2009)—4.65–4.72 mg/100 g and Lombardi‐Boccia et al. (2005)—3.94–4.75 mg/100 g. Similar amounts of this element in beef were found by Czerwonka and Szterk (2015)—3.5–6.9 mg/100 g.

In turn, Gerber et al. (2009) showed a lower content of phosphorus in beef elements (162–168 mg/100 g) than the level of this element determined in the analyzed muscles in the author's own research.

Lombardi‐Boccia et al. (2005) found similar or slightly higher copper contents (0.04–0.09 mg/100 g) in particular elements of beef.

## 5 CONCLUSIONS

On the content of minerals in muscles obtained from beef carcasses, the statistically significant impact was found in the case of slaughter type for such elements as follows: K, P and, Na. Higher content of phosphorus, potassium, and sodium was found in muscles of heifers and bulls from standard slaughter, compared with the amount of these elements determined in muscles of cattle from kosher slaughter. The process of koshering (soaking in brine) causes approximately 10‐fold increase in the amount of sodium in beef, regardless of the muscle or gender of cattle. In turn, statistically significant impact of cattle sex was confirmed only in the case of iron and molybdenum content in beef. The statistically significant impact of muscle was confirmed only in the case of zinc content. Higher amounts of this element were determined in the MS muscle of cattle, regardless of the type of slaughter.

## CONFLICT OF INTEREST

The authors declare that they have no conflict of interest.

## ETHICAL STATEMENT

This study does not involve any human nor animal testing.
